# Feasibility and accuracy of bedside transthoracic echocardiography in diagnosis of acute proximal aortic dissection

**DOI:** 10.1186/s12947-015-0008-5

**Published:** 2015-03-25

**Authors:** Dorota Sobczyk, Krzysztof Nycz

**Affiliations:** The Department of Interventional Cardiology, John Paul 2nd Hospital, Pradnicka 80, 31 202 Krakow, Poland

**Keywords:** Acute aortic syndromes, Acute aortic dissection, Aortic aneurysm, Transthoracic echocardiography, Bedside echocardiography

## Abstract

**Study objective:**

The purpose of the present study was to establish the accuracy of transthoracic echocardiography (TTE) in diagnosis of acute type A aortic dissection in comparison to computed tomography (CT), with reference to the intraoperative image.

**Methods:**

The retrospective analysis included 178 patients referred to the cardiac surgery unit in our center due to acute type A dissection between 01-01-2008 and 31-12-2013, who underwent both TTE and CT. Intraoperative image was considered as a reference.

**Results:**

Statistical analysis did not show any significant differences between computed tomography and echocardiography in the detection of the proximal aortic dissection. In patients with aortic valve abnormalities, procedure of choice was replacement by a composite graft (77,59%), whereas patients with a normal image of aortic valve were more likely to have the valve sparing procedure (50,88%). The R-Spearman statistics shows a strong positive correlation between maximum diameter of ascending aorta measured by TTE and CT (cc 0.869) and TTE and intraoperative measurement (cc 0.844).

**Conclusion:**

Our data confirm that transthoracic echocardiography is a reliable method for diagnosis of proximal aortic dissection. TTE provides a reliable value of maximum diameter of the ascending aorta in comparison to both CT and direct intraoperative measurement. Moreover, transthoracic echocardiography gives the additional information that influences the operative technique of choice and identifies the high-risk patients (cardiac tamponade, severe aortic dilatation, severe aortic regurgitation). Our retrospective analysis confirms the pivotal role of TTE in the evaluation of the patients with suspected proximal aortic dissection in emergency room setting.

## Introduction

Acute aortic dissection is a life-threatening condition that requires a prompt diagnosis and definite management [1,2]. Acute thoracic aortic dissection involving the ascending aorta (type A by Stanford classification, proximal aortic dissection) is an undoubtful indication for emergent surgical intervention [2,3]. The well documented mortality rise of 1-2% for each hour following type A dissection highlights the importance of the rapid, easily accessible, noninvasive diagnostic method [4-6]. Computed tomography (CT) is considered a gold standard that enables to visualize the entire aorta and to distinguish among the different types of acute aortic syndromes [3,7,8]. However, this technique is not always available, requires transferring the patient to the CT lab and often generates a significant delay in treatment. Transthoracic echocardiography (TTE) given its rapidity, availability, portability and safety seems an ideal imaging technique for the initial evaluation of the patients with suspected proximal aortic dissection.

The purpose of our present study was to assess the accuracy of TTE in diagnosis of acute type A aortic dissection in comparison to computed tomography, with reference to the intraoperative image (actual surgical findings).

## Methods

We retrospectively examined medical data of patients transferred to our center due to suspected acute aortic dissection between 01-01-2008 and 31-12-2013.

The inclusion criteria were: referral for an urgent surgery due to the proximal aortic dissection (type A according to Stanford classification), available results of both CT and bedside TTE. The study group consisted of 178 consecutive patients (47 female/131 male, mean age 58,69 ± 12,61 yrs), qualified for the urgent cardiac surgery. Eventually, among 178 patients who met the inclusion criteria, the cardiac surgery was finally conducted in 172 patients (1 patient refused the operation and died, the other 5 patients underwent cardiac arrest and died before being transferred to the operating theatre). 28 patients died in the operating theatre, during the cardiac surgery and 33 patients died in the postoperative course, during in-hospital stay (before discharge). The study protocol was approved by the Local Ethical Committee of John Paul 2^nd^ Hospital in Cracow.

The transthoracic echocardiographic examinations were performed in the emergency unit using a Vivid I (GE) or a CX 50 (Philips) ultrasound unit and a S3 3.5-5 MHz transducer. All patients transferred to our center with suspected aortic dissection, were examined by one of the experienced echocardiographers, being a member of the on-call team (including 5 cardiologists). The echocardiographic diagnosis of the proximal aortic dissection was based on the demonstration of the presence of an intimal dissection flap in the ascending aorta dividing the aorta into the two lumina. The maximum diameter of the ascending aorta was measured in diastole using the leading edge-to-leading edge convention. The following echocardiographic parameters were also evaluated: left ventricular ejection fraction, pericardial effusion (and cardiac tamponade features), aortic valve morphology (number of cusps, calcifications), hemodynamically significant aortic valve regurgitation, other valvular disease. Computed tomography in most cases (90%) was performed using Siemens Sensation 64 with intravenous contrast injection.

Medical records were analyzed independently by both authors and in each case a consensus was achieved. No ambiguous examinations (CT and TTE) was recorded, all results were clearly stated in a dichotomic manner: dissection was present or not.

Intraoperative finding (actual surgical view reported by operating surgeon: aneurysm, diameter of aorta, dissection flap, intramural hematoma) was considered a reference gold standard.

Statistics was done using STATISTICA v 8.0 software for all analyses. The chi square test was used to compare the accuracy of both diagnostic methods (transthoracic echocardiography and computed tomography) in diagnosis of proximal aortic dissection. Given the lack of a normal probability distribution, correlation of the maximum diameter of ascending aorta measured with different diagnostic modalities was evaluated using R-Spearman statistics. The Bland-Altman test was performed to assess the bias between the techniques.

## Results

The retrospective analysis included 178 patients referred to the cardiac surgery unit in our center due to acute type A dissection between 01-01-2008 and 31-12-2013, who underwent both TTE and CT. Because intraoperative finding was considered a reference for the presence of aortic dissection, 6 patients who died without cardiac surgery, were excluded from the final analysis. The demographic and medical data of the study population are shown in Table 1.

**Table 1:** **Demographic and medical data of studied population**

**Variable**	
Age ± SD [*yrs*]	58,69 ± 12,61
Women/men *n (%)*	47 (26,4%)/131 (73,6%)
Cardiogenic shock *n (%)*	131 (73,6%)
Marfan syndrome *n (%)*	2 (1,12%)
Trauma prior to admission *n (%)*	1 (0,56%)
History of the cardiac or vascular surgery *n (%)*	11 (6,18%)
Prior AVR	5 (2,81%)
Prior CABG	4 (2,25%)
Prior abdominal aneurysm repair	2 (1,12%)
Type of cardiac surgery *n (%)*	
Alloplasty of ascending aorta using composite aortic graft (Bental de Bono procedure)	101 (58,72%)
Alloplasty of ascending aorta sparing aortic valve	63 (36,63%)
Alloplasty of aortic arch	36 (20,93%)
Aortic valve plasty	5 (2,91%)
CABG	10 (5,81%)
Stent-graft deployment in the descending aorta	2 (1,16%)
The brachio-cephalic trunk plasty	2 (1,16%)
MVR	1 (0,58%)
Deaths *n (%)*	69 (38,76%)
Preoperative	6 (3,37%)
Intraoperative	30 (16,85%)
Postoperative (before discharge)	33 (18,54%)
Echocardiographic findings	
Aortic dissection *n (%)*	159 (89,32%)
Severe aortic dilatation (max. diameter ≥ 60 mm) *n (%)*	60 (34,48%)
LVEF ± SD [%]	49,62 ± 11,97
LVEF ≤ 35% *n (%)*	32 (18,39%)
Regional wall motion abnormalities *n (%)*	55 (31,6%)
Cardiac tamponade *n (%)*	48 (26,97%)
Bicuspid aortic valve *n (%)*	9 (5,06%)
Calcification of the tricuspid aortic valve *n (%)*	10 (5,62%)
Moderate/severe aortic regurgitation *n (%)*	53 (29,78%)
Calcific mitral stenosis *n (%)*	1 (0,56%)

**Figure 1:**
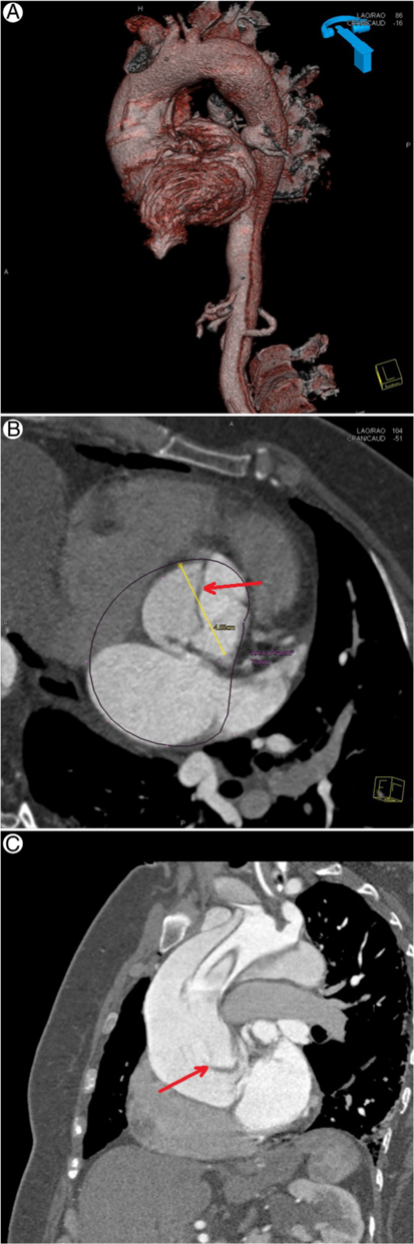
**Computed tomography showing dilated ascending aorta with flap of proximal dissection. A**. Computed tomography – 3-D reconstruction image: dilated ascending aorta is visible with a flap of proximal dissection along the distance of visualized vessel. **B**. Computed tomography – transverse plane zoom image: at the level of aortic root a true and false lumina of the vessel are visible, separated by a flap of dissection. **C**. Computed tomography – tangenital plane: showing ascending aorta and partially false lumen with an intimal flap of proximal aortic dissection.

**Figure 2:**
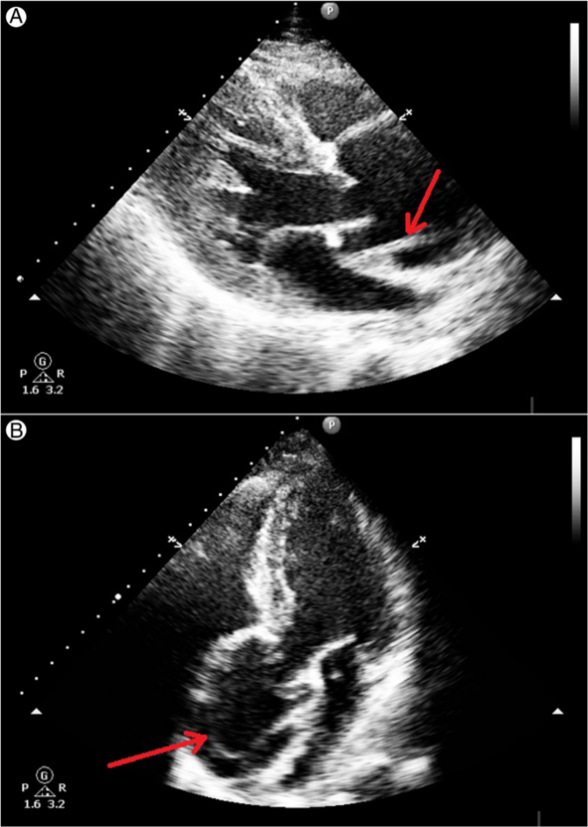
**Transthoracic echocardiography showing linear echo of intimal tear in dilated aortic root above aortic valve level. A**. Transthoracic echocardiography – parasternal long axis view: linear echo of intimal tear is seen just above aortic valve in systole. **B**. Transthoracic echocardiography – apical 5-chamber view: linear echo of intimal flap is visible in dilated aortic root above aortic valve level.

**Figure 3:**
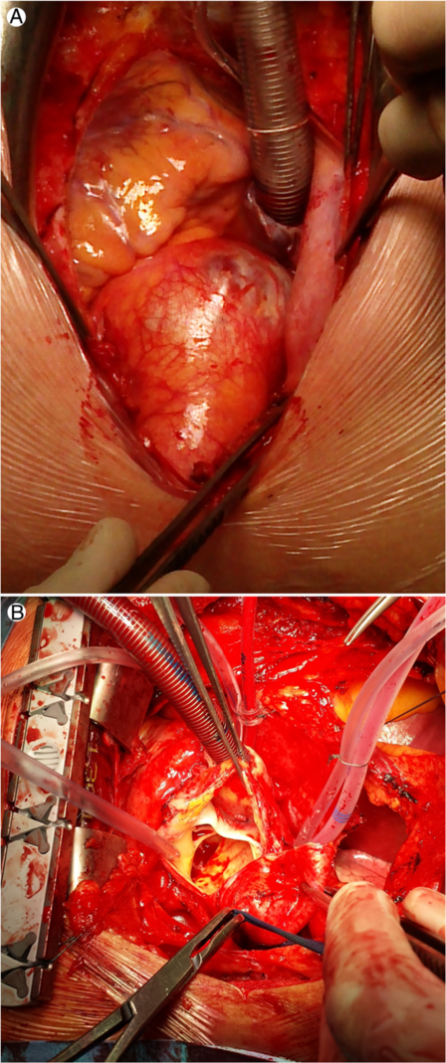
**Intraoperative image showing dilated ascending aorta. A**. Intraoperative image: dilated ascending aorta is seen in an operative field. **B**. Intraoperative image: view from the operating field after cross-section of ascending aorta, flap of dissection separating a true from a false lumen is visible.

Computed tomography demonstrated type A aortic dissection in all 172 patients (100%) of the study population (Figure 1). The presence of the intimal dissection flap in the ascending aorta was noted by TTE in 159 patients (92,44%) (Figure 2). Intraoperative inspection showed aortic dissection of type A according to Stanford classification in 171 patients (99,42%) (Figure 3). In only one patient after blunt chest trauma (traffic accident), with positive CT result (but no intimal flap seen on TTE), aortic dissection was not confirmed intraoperatively. The echocardiographic data of the study population are shown in Table 2. Statistical analysis with chi square test did not show any statistically significant differences between computed tomography and echocardiography in the detection of the proximal aortic dissection (p 0,000). Additionally, bedside transthoracic echocardiography revealed the concomitant abnormalities, i.e. bicuspid aortic valve, AV calcifications, moderate/severe aortic incompetence or cardiac tamponade. All the echocardiographic findings were confirmed intraoperatively and influenced the treatment strategy. In the patients with any aortic valve abnormalities (bicuspid aortic valve, AV calcifications, significant aortic regurgitation) procedure of choice was replacement by a composite graft (77,59% vs 49,12%, p = 0,001), whereas the patients with normal aortic valves were significantly more likely to have the valve sparing surgery (50,88% vs 22,41%, p = 0,001).

**Table 2:** **Correlation of the aortic diameter (maximum size) obtained by the different techniques (TTE/CT/intraoperative view)**

**Correlation between the methods**	**Max. diameter ± SD** ***[mm]***	**Mean difference ± SD** ***[mm]***	**r**	**p**
TTE vs CT	56,46 ± 10,79 vs 58,76 ± 11,96	3,89 ± 3,88	0,869	<0,001
TTE vs intraop	56,46 ± 10,79 vs 62,02 ± 15,30	4,22 ± 5,26	0,844	<0,001
CT vs intraop	58,76 ± 11,96 vs 62,02 ± 15,30	3,94 ± 5,59	0,838	<0,001

**Figure 4:**
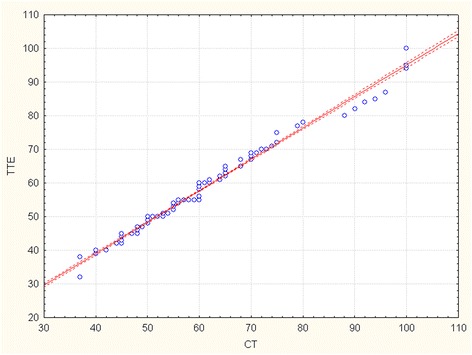
**Scatterplot showing the correlation between transthoracic echocardiography and computed tomography measurements for the maximal dimensions of the ascending aorta.**

**Figure 5:**
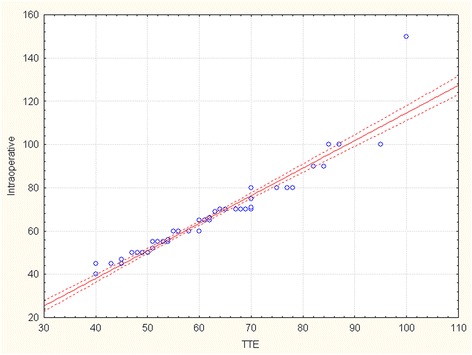
**Scatterplot showing the correlation between transthoracic echocardiography and intraoperative measurements for the maximal dimensions of the ascending aorta.**

**Figure 6:**
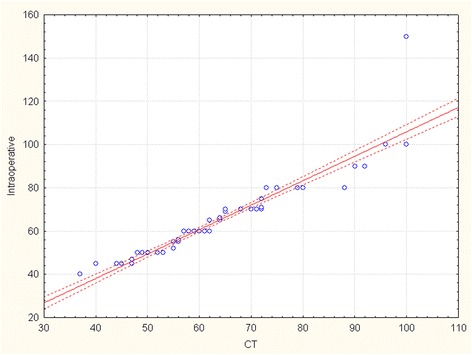
**Scatterplot showing the correlation between computed tomography and intraoperative measurements for the maximal dimensions of the ascending aorta.**

The R-Spearman statistics was used to compare aortic diameters assessed by TTE with CT and intraoperative measurement (Table 2). There is a strong positive correlation between maximum diameter of ascending aorta measured by TTE and CT (correlation coefficient 0.869, p < 0,001). Maximum aortic diameter assessed by TTE correlates as well with direct intraoperative measurement (correlation coefficient 0.844, p < 0,001). Correlation curves and scatterplots for analysis of agreement between the measurements of maximum aortic diameter performed by 2 different techniques are shown in Figure 4 (TTE vs CT), Figure 5 (TTE vs intraop) and Figure 6 (CT vs intraop).

## Discussion

This retrospective analysis of 178 patients shows that transthoracic echocardiography is not inferior to computed tomography in diagnosis of acute type A aortic dissection with reference to the intraoperative finding. Our data confirm the efficacy and accuracy of TTE in evaluating the maximum size of ascending aorta in the subset of emergency patients in comparison to both CT and direct intraoperative measurement. Observed small differences between the methods of measurement did not reach statistical significance and were associated with the different measurement techniques. First of all, the maximum diameter of ascending aorta perpendicular to the axis of blood flow was measured, that can differ given the area of imaging in different imaging modalities. Additionally, in both CT and intraoperative measurements, the maximum external size of the ascending aorta was assessed, whereas in TTE the leading edge-to-leading edge convention was used. In severely dilated aneurysms (with diameter excessing 8 cm; see Results) TTE underestimated the maximum aortic size, however it did not influence the patients management.

Moreover, transthoracic echocardiography gives the additional information on aortic valve morphology and functional status (i.e. bicuspid aortic valve, severe AV calcifications, significant aortic regurgitation) that are often crucial for the operative technique choice. In our study, patients with any aortic valve abnormalities detected by TTE (33,33%), were significantly more likely to undergo a composite graft replacement (77,59%). In patients with confirmed aortic dissection, TTE identifies also high-risk features, such as severe proximal aortic dilatation (34,48% in our group), cardiac tamponade (26,97%), regional wall motion abnormalities (31,6%) and severely impaired left ventricular ejection fraction (18,39%).

Our findings support the pivotal position of TTE in the management of the patients with clinically suspected acute type A aortic dissection [9-14]. Previously, TTE has been considered limited in the diagnosis of acute aortic dissection, with sensitivity 78-90% and specificity 87-96% for type A dissection [7,14]. However, these data originate from relatively old studies and most probably do not reflect the reality in the era of new echocardiographic technology (i.e. excellent 2D resolution or harmonic imaging). More recent data reported by Cecconi et al. [15] in 2012, where TTE was compared to CT in suspected acute aortic dissection, show high sensitivity and specificity (97 and 100%, respectively) in patients with optimal image quality. In our patients (studied population) TTE reached sensitivity of 99,4% in diagnosis of acute type A dissection, although these data originate from a highly selected population of patients (that have been transferred to our center with an already established suspicion of aortic dissection, in almost 50% after CT diagnosis). However, the additional advantage of our study is the comparison of both noninvasive diagnostic methods (CT and TTE) with the intraoperative finding (that was considered a reference value).

It should be remembered that TTE has several limitations, like poor visualization of intramural hematoma, the upper segment of ascending aorta or entry tear location. Using a multi-plane approach (thorough step-by-step examination using all available TTE views) allows visualization of the entire ascending aorta in majority of patients. Contrast enhancement substantially improves TTE in the diagnosis of aortic dissection, that was proved by Evangelista et al. [16] In that study sensitivity and specificity of conventional TTE significantly increased after contrast enhancement.

Computed tomography remains a gold standard imaging modality in the diagnosis of aortic dissection. However still in the guidelines, is often not immediately available, impossible to be done in a bedside manner, and always results in a significant delay to treatment. It also requires an intravenous contrast administration and a transfer of a potentially high-risk patient to the CT laboratory. TTE plays an important role in the first-line emergency assessment of patients with clinically suspected proximal aortic dissection. This method is rapid, noninvasive, safe, readily available, portable, utilizes no radiation and does not influence the hemodynamic state or disturb preparation for the urgent cardiac surgery. That value of TTE may be limited in patients with poor acoustic window (obesity, pulmonary emphysema, chest wall abnormalities, mechanical ventilation), despite we did not observe such in our study. EAE clinical recommendations emphasize the role of TTE as the initial imaging modality when aortic dissection is clinically suspected. The current joint practical guidelines of the cardiac, cardiothoracic, anesthesiology, radiology and vascular societies, recommend the selection of a specific imaging modality based on an immediate availability (class I, level of evidence C) [3]. Negative initial aortic imaging with TTE always requires a second imaging study with higher negative predictive value, e.g. CT or transesophageal echocardiography, to rule out the dissection, when clinically suspected (class I, level of evidence C) [3].

Previously, Meredith and Masani [17] in their review article underline the role of a rapid TTE assessment in the emergency setting. The authors propose the diagnostic algorithm for suspected aortic dissection with an immediate TTE as a first-line imaging technique. They recommend that patients with clear evidence of aortic dissection and high risk features (cardiac tamponade, severe aortic dilatation, severe aortic regurgitation) should be immediately transferred to the operating theatre. This approach minimizes delays and lowers preoperative mortality.

In fact, in our center the same diagnostic algorithm has been used for at least 10 years. TTE is a first-line technique of choice in all the patients transferred to our department with a suspicion of acute proximal aortic dissection regardless CT results. The patients with a certain echochardiographic diagnosis of type A aortic dissection are transferred directly to the operating theatre, usually without any additional diagnostic tests (if not required by the surgeon). TTE does not visualize the entire aorta nor provides comprehensive information about the extent of dissection membrane. However, in unstable high-risk patients (e.g. cardiogenic shock), immediate cardiac surgery remains life-saving procedure. In those cases with diagnosis established by TTE, surgery is performed based on limited echocardiographic data to reduce mortality related to time delay. In our centre, computed tomography or preoperative transesophageal echocardiography (TEE) is performed in stable patients and those with a negative TTE result. Given the last 5 years, about 30% of all the cardiac surgery procedures for acute proximal aortic dissection were conducted based on echocardiographic diagnosis, with 100% intraoperative confirmation. We believe that this approach facilitates the rapid diagnosis of acute type A aortic dissection and shortens the delay to definite treatment.

## Conclusion

1. Transthoracic echocardiography is a reliable method for diagnosis of proximal aortic dissection.
2. TTE provides a reliable value of maximum diameter of the ascending aorta in comparison to both CT and direct intraoperative measurement.
3. Moreover, transthoracic echocardiography gives the additional information that influences the operative technique choice (aortic valve abnormalities) and identifies the high-risk patients (cardiac tamponade, severe aortic dilatation, severe AR).
4. Our retrospective analysis emphasizes the pivotal role of TTE in the emergency evaluation of the patients with suspected proximal aortic dissection.

### Limitations

The limitation of the study is a retrospective analysis. All the patients who underwent cardiac surgery for acute proximal aortic dissection based on TTE without CT verification (i.e. about 30% of all the patients between 01-01-2008 and 31-12-2013), were excluded from the analysis. We examined highly selected group of the patients transferred to our center from district hospitals with a clinical suspicion of aortic dissection, often confirmed by CT or initial TTE, that undoubtedly favors echocardiography in the diagnosis of dissection.

## Abbreviations

TTE: Transthoracic echocardiography
CT: Computed tomography
TEE: Transesophageal echocardiography
